# Effect of tobacco use on cadmium accumulation in the oral keratinized mucosa

**DOI:** 10.1186/s12903-024-04001-6

**Published:** 2024-02-20

**Authors:** Samed Satir, Dogan Ilgaz Kaya, Sumeyye Celik Ozsoy

**Affiliations:** 1 Faculty of Dentistry, Oral and Maxillofacial Radiology, Karamanoglu Mehmetbey University, Karaman, 70200 Turkey; 2 Faculty of Dentistry, Oral and Maxillofacial Surgery, Karamanoglu Mehmetbey University, Karaman, Turkey 70200

**Keywords:** Oral mucosa, Cadmium, Oral cancer, Oral keratinized mucosa

## Abstract

**Background:**

This cross-sectional study aimed to evaluate the effect of tobacco use on the accumulation of cadmium (Cd), a carcinogenic element, in the oral keratinized mucosa (OKM).

**Methods:**

OKM samples were obtained by standard punch biopsy from nonsmokers (*n* = 19) and smokers (*n* = 21). Cd analysis was performed using inductively coupled plasma optical emission spectroscopy (ICP-OES). The calibration curve R2 values for three wavelengths (214,439, 226,502, and 228,802 nm) were at the level of 0.9999. The frequency of consumption of foods that are Cd sources, such as seafood, rice, and vegetables, was assessed in all patients. The age, sex, and nutritional habits of all patients and the frequency of tobacco consumption by smokers were recorded. The independent t-test, Mann–Whitney U test, Fisher’s exact test, and Spearman correlation test were used for the statistical analyses, and *p* < 0.05 was considered significant.

**Results:**

Although the Cd levels in nonsmokers were higher than those in smokers, no statistically significant difference was found (*p* > 0.05). In smokers, a positive correlation was found between age and Cd level (*r* = 0.574, *p* = 0.006). No significant relationship was found between the groups in terms of nutrition or between the frequency of tobacco consumption and Cd accumulation.

**Conclusion:**

The OKM may not have the characteristic cumulative accumulation in terms of toxic elements. Changes in the turnover rate, keratinization, and apoptotic mechanisms in the OKM with the thermal/chemical effects of tobacco may be responsible for the difference in Cd accumulation.

**Trial registration number:**

TCTR20230206001/06 Feb 2023 (TCTR: Thai Clinical Trials Registry).

## Background

Cadmium (Cd) is a toxic element, and Cd concentrations increase in industrial areas, highly polluted areas, tobacco products, seafood, and foods such as rice grown in soil contaminated with Cd [1–3].

Cd accumulates mostly in the kidney, liver, bone, and brain [1, 2]. Cd, which can cause toxic effects when accumulated in the tissues, competes with the trace elements Zn and Fe, which are necessary for metabolism, and Cd levels decrease if the levels of these two elements are sufficient in the body [3].

Cd, which is classified as a toxic and a carcinogenic element, plays a role in the etiology of many cancer types [4–6]. Cd, which is believed to be effective in the development of oral cancer, is 1 of > 3,000 chemical components in tobacco products. Cd influences the formation of oral cancer because it changes the apoptotic mechanism in the mucosa [7–10]. In addition, the Cd level was higher in various biological samples, such as hair, blood, and dental calculus, taken from patients with oral cancer than in those from healthy individuals [3, 8, 11, 12]. Apart from oral cancers, tobacco products (smoke or smokeless) have an important role in the etiology of premalignant white lesions such as oral leukoplakia, which may show hyperkeratosis and epithelial dysplasia [13, 14].

The oral mucosa is the tissue with which tobacco products and cigarette smoke first come into contact [15]. Cd in tobacco can also accumulate in the oral mucosa through epithelial permeability because of this contact. Thus, this study aimed to evaluate changes in Cd accumulation in the oral keratinized mucosa (OKM) due to tobacco use.

## Methods

### Sample size

The sample size was calculated using the G*Power 3.1.9 program. Zhang et al. [12] conducted a Cd concentration analysis study using dental calculus samples from individuals with oral squamous cell cancer (OSCC) and healthy individuals (controls). The mean Cd levels in the OSCC and healthy groups were 464.0 ± 1 26.61 parts per billion (ppb) and 224.5 ± 58.55 ppb, respectively. According to these results, the effect size was calculated as d = 2.428. For 95% statistical power and 0.05 margin of error, the minimum sample size planned to be included in the study was 12 patients (OSCC group, *n* = 6; control group, *n* = 6).

### Samples collection

**Fig. 1:**
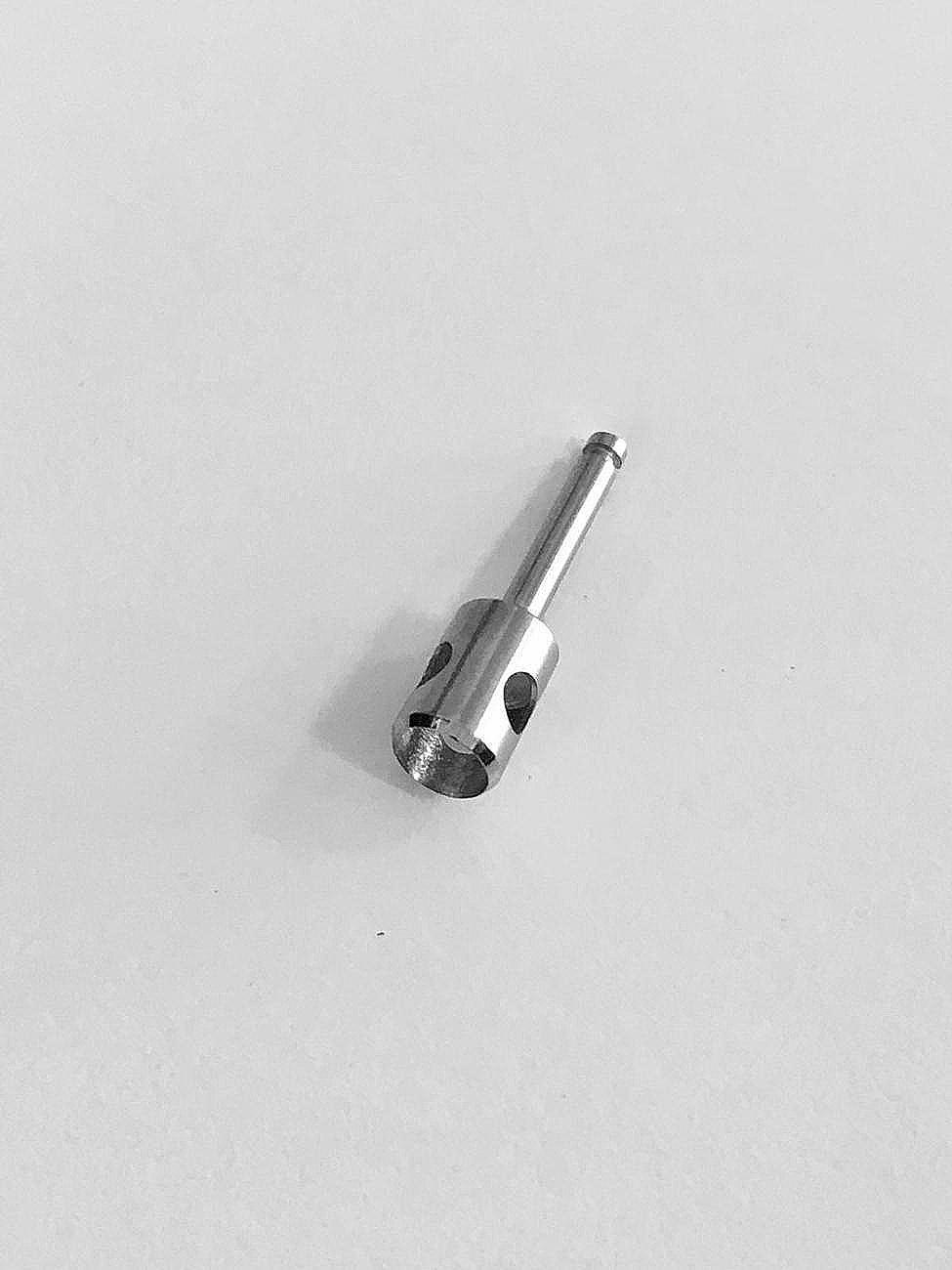
Sterilizable Punch biopsy apparatus

Patients aged > 18 years who applied to Karamanoğlu Mehmetbey University Faculty of Dentistry for implant-supported prosthesis between January 2023 and June 2023 were included in this cross-sectional study. Based on previous similar studies [12, 16], individuals with a history of cancer, radiotherapy/chemotherapy treatment, chronic inflammatory diseases, diabetes and/or hypertension, and gastrointestinal absorption disorders (such as Crohn’s disease, celiac disease, and bowel syndrome) were excluded from the study. Approximately 3 months after the implant surgery, the alveolar OKM on the implant was removed to connect the implants with the oral cavity and construct the dental prosthesis, and this tissue became medical waste. Before the OKM was removed, patients rinsed their mouths with saline solution for 15 s. The OKM obtained was stored at − 18 °C until Cd concentration analysis. A sterilizable punch biopsy apparatus was used to standardize the obtained samples (Fig. 1).

### Patient classification

OKM samples were divided into two groups: samples from patients who had never used tobacco products (*n* = 19) and those who still used tobacco products (*n* = 21). Individuals who consumed tobacco products during a certain period of their lives and gave up on this habit were excluded from the study because their inclusion may lead to contradictory results when showing tobacco product-induced Cd accumulation in the OKM. The age and sex of the study participants were recorded. The frequency of consumption (none, once a month, once a fortnight, once a week, 2 days a week, and 3–4 days a week) of seafood, rice, and tuberous vegetables (potatoes, carrots, radishes, celery, etc.) was collected. Smokers were asked for the number of years they had consumed tobacco products (1–3, 4–7, 8–10, 11–15, 16–20, and > 20 years) and the number of pieces consumed daily (1–5, 6–10, 11–20, 20–40, and > 40 pieces). In addition, the study participants were also asked whether their jobs could expose them to various heavy metal dusts (mercury, arsenic, cobalt, etc.) or whether they reside near an environment such as a factory or mine site.

### Cd concentration determination

Because the thickness of the samples taken at the same diameter using the standard punch biopsy apparatus may vary, the OKM samples were weighed. The sample weight information was used to calculate the exact Cd levels after Cd concentration determination.

**Fig. 2:**
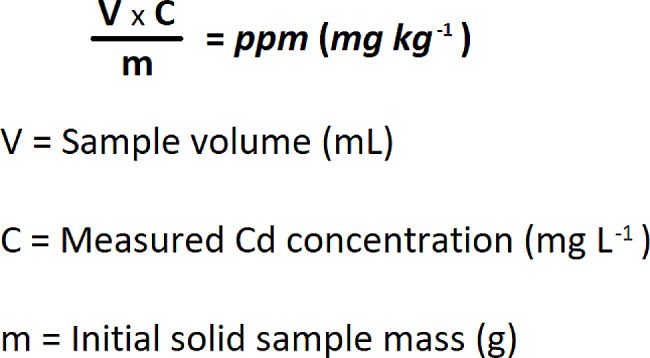
The formula for calculating the exact Cd value of the samples

OKM samples (mean, 27 mg) were weighed in a tube (50 mL). Then, 2 mL of 65% nitric acid (HNO_3_) was added. The tube was sealed with a cup and set off overnight (approximately 16 h). Then, 1 mL of HNO_3_ and 1 mL of 30% hydrogen peroxide (H_2_O_2_) were added. The temperature of the tube was increased to 70 °C in 30 min and maintained at 70 °C for 4 h. The solution obtained was added with water to make 5 mL. The inductively coupled plasma optical emission spectroscopy (ICP-OES) device (Agilent Technologies 720, CA, USA) in the Karamanoglu Mehmetbey University Scientific and Technological Research Application and Research Center (BILTEM) laboratory was used to determine the Cd concentrations in the OKM. To determine the Cd level, three wavelengths (214,439, 226,502, and 228,802 nm) were evaluated. The calibration curve R^2^ value for each wavelength was at the level of 0.9999. The ICP-OES device was used for three concentration measurements. Measurements are expressed in ppb, and negative values are accepted as zero. The highest value obtained from the three measurements made for the most suitable wavelength selected was set as the Cd level value. The values read and recorded in the device were the concentrations of the solution obtained by dissolving the samples; therefore, the exact sample value was calculated according to the formula in Fig. 2. The final volume in the thawing process was 5 mL, and the initial mass (gram) of each solid sample was determined. The exact Cd levels in the samples were expressed in parts per million (ppm).

### Statistical analysis

Categorical variables were presented as frequencies (n) and percentages (%). Continuous data were represented as mean ± standard deviation or median with percentiles (interquartile range [IQR], 25th − 75th percentiles) based on the distribution, which was controlled by the Shapiro–Wilk test. Fisher’s exact test was used to determine the relationship between the categorical variables. The Mann–Whitney U test and independent t-test were used for nonparametric and parametric comparisons of numeric data between the study groups, respectively. The Spearman correlation test was applied to determine the association among the study parameters. Data were analyzed using IBM SPSS Statistics for Windows, version 23.0 (IBM Corp., Armonk, NY, USA). A two-sided p-value of < 0.05 was considered statistically significant.

## Results

The mean age of the study participants was 52.03 ± 14.48 years, and 57.5% were men. Moreover, 61.9% of the patients had been smoking for ≥ 21 years, 52.4% of the smokers smoked between 11 and 20 cigarettes/day, and 16 (40%) patients stated that they consumed seafood once a month, 17 (42.5%) consumed rice once a week, and 22 (55%) consumed tuberous vegeTable 3, and 4 days a week. The median OKM weight was 0.024 g (IQR, 0.013–0.04), and the median Cd level was 111,367 ppm (IQR, 55,607–191,883) (Table 1).

**Table 1:** Patient characteristics

	All patients (*n* = 40)
Age (years), mean ± SD	52,03 ± 14,48
Gender, n(%)	
Female	17(42,5)
Male	23(57,5)
Smoking status, n(%)	
Non-smokers	19(47,5)
Smokers	21(52,5)
Duration of tobacco consumption, n(%)	
4–7 years	2(9,5)
8–10 years	1(4,8)
11–15 years	5(23,8)
21 years and more	13(61,9)
Piece of daily cigarette consumption, n(%)	
1–5	1(4,8)
6–10	3(14,3)
11–20	11(52,4)
21–40	6(28,6)
Seafood consumption frequency, n(%)	
Never	10(25)
One day a month	16(40)
once every two weeks	7(17,5)
One day per week	5(12,5)
3–4 days a week	2(5)
Rice consumption frequency, n(%)	
Never	1(2,5)
One day a month	6(15)
once every two weeks	3(7,5)
One day per week	17(42,5)
3–4 days a week	13(32,5)
Tuberous vegetables consumption frequency, n(%)	
Never	1(2,5)
One day a month	2(5)
once every two weeks	2(5)
One day per week	13(32,5)
3–4 days a week	22(55)
OKM weight (gram), median(IQR)	0,024(0,013 − 0,04)
Cd level, median(IQR)	111,367(55,607 − 191,883)

No significant difference was found between the patients’ mean age (*p* = 0.326) and consumption frequency of seafood (*p* = 0.132), rice (*p* = 0.592), and tuberous vegetables (*p* = 0.580) according to the smoking status. The OKM was heavier in smokers than in nonsmokers, and the Cd level was lower in smokers, but this was not significantly different (*p* = 0.236 and *p* = 0.708, respectively) (Table 2).

**Table 2:** Patient characteristics according to smoking status

	Non-smokers (*n* = 19)	Smokers (*n* = 21)	p
Age (years), mean ± SD	54,42 ± 15,84	49,86 ± 13,15	0,326
Gender, n(%)			
Female	14(73,7)	3(14,3)	**< 0,001**
Male	5(26,3)	18(85,7)	
Seafood consumption frequency, n(%)			
Never	8(42,1)	2(9,5)	0,132
One day a month	7(36,8)	9(42,9)	
once every two weeks	2(10,5)	5(23,8)	
One day per week	2(10,5)	3(14,3)	
3–4 days a week	0(0)	2(9,5)	
Rice consumption frequency, n(%)			
Never	0(0)	1(4,8)	0,592
One day a month	2(10,5)	4(19)	
once every two weeks	2(10,5)	1(4,8)	
One day per week	10(52,6)	7(33,3)	
3–4 days a week	5(26,3)	8(38,1)	
Tuberous vegetables consumption frequency, n(%)			
Never	0(0)	1(4,8)	0,580
One day a month	1(5,3)	1(4,8)	
once every two weeks	2(10,5)	0(0)	
One day per week	7(36,8)	6(28,6)	
3–4 days a week	9(47,4)	13(61,9)	
OKM weight (gram), median(IQR)	0,022(0,009 − 0,039)	0,035(0,014 − 0,041)	0,236
Cd level, median(IQR)	140,948(50,786 − 227,618)	108,685(60,377 − 176,811)	0,708

Independent t-test, Mann-Whitney U test, Fisher’s Exact test

No significant differences were found in the OKM weight or Cd levels by sex in all patients (*n* = 40) and smoking status (*p* > 0.05). Although the Cd level was higher in men than in women, no statistically significant difference was found (*p* > 0.05). The OKM was heavier in male than in female nonsmokers; however, it was heavier in female than in male smokers. However, no statistically significant difference was found between the two groups (*p* > 0.05) (Table 3).

**Table 3:** Cd level and OKM weight according to gender

All patients (*n* = 40)	Gender		p
	Female (*n* = 17)	Male (*n* = 23)	
OKM weight (gram)	0,024(0,014 − 0,038)	0,023(0,012 − 0,043)	0,498
Cd level, median(IQR)	106,175(50,786 − 227,618)	111,645(64,761 − 181,723)	0,745
**Non-smokers (** ***n*** ** = 19)**	**Female** **(** ***n*** **= 14)**	**Male** **(** ***n*** **= 5)**	
OKM weight (gram)	0,02(0,009 − 0,035)	0,023(0,009 − 0,059)	0,622
Cd level, median(IQR)	130,516(50,786 − 269,057)	157,698(80,396 − 175,909)	0,964
**Smokers (** ***n*** ** = 21)**	**Female** **(** ***n*** **= 3)**	**Male** **(** ***n*** **= 18)**	
OKM weight (gram)	0,038(0,035 − 0,046)	0,026(0,013 − 0,041)	0,412
Cd level, median(IQR)	60,377(38,654 − 106,175)	111,367(64,761 − 181,723)	0,221

Mann-Whitney U test

No significant correlation was found between age, OKM weight, and Cd levels in all patient groups (*n* = 40) and control group (*n* = 19) (*p* > 0.05). In smokers (*n* = 21), a moderate positive correlation was found between age and Cd levels (*r* = 0.574; *p* = 0.006). A negative correlation was found between age and OKM weight (Table 4).

**Table 4:** Correlation between age and Cd level and OKM weight

All patients (*n* = 40)	Age	
	r	p
OKM weight (gram)	-0,160	0,325
Cd level	0,208	0,198
**Non-smokers (** ***n*** ** = 19)**		
OKM weight (gram)	-0,006	0,982
Cd level	-0,127	0,606
**Smokers (** ***n*** ** = 21)**		
OKM weight (gram)	-0,287	0,208
Cd level	0,574	**0,006**

Spearman correlation test

No significant correlation was found between the frequency of consumption of seafood, rice, and tuberous vegetables and OKM weight and Cd levels in all patients (*p* > 0.05). A moderate positive correlation was noted between the frequency of consumption of tuberous vegetables and Cd levels in nonsmokers; however, this relationship was not significant (*p* = 0.074) (Table 5).

**Table 5:** Correlation between food consumption frequency and Cd level and OKM weight

All patients (*n* = 40)	Seafood		Rice		Tuberous vegetables	
	r	p	r	p	r	p
OKM weight (gram)	0,196	0,226	-0,069	0,672	-0,015	0,925
Cd level	0,024	0,881	-0,059	0,718	0,200	0,215
**Non-smokers (** ***n*** ** = 19)**						
OKM weight (gram)	0,119	0,627	-0,042	0,865	-0,086	0,726
Cd level	0,045	0,855	0,041	0,867	0,420	0,074
**Smokers (** ***n*** ** = 21)**						
OKM weight (gram)	0,151	0,514	-0,121	0,601	-0,037	0,874
Cd level	0,191	0,407	-0,126	0,587	-0,015	0,948

Spearman correlation test

A positive moderate correlation was found between the duration of smoking and Cd levels in smokers; however, it was not significant (*p* = 0.062). No significant correlation was noted between daily tobacco product consumption and OKM weight and Cd levels (*p* > 0.05) (Table 6).

**Table 6:** Correlation between smoking duration-number of daily cigarette consumption in smokers and Cd level and OKM weight

Smokers (*n* = 21)	Smoking duration		Piece of daily cigarette consumption	
	r	p	r	p
OKM weight (gram)	-0,144	0,534	0,093	0,690
Cd level	0,414	0,062	0,144	0,534

Spearman correlation test

## Discussion

Cd accumulates in the kidney, liver, and bone [1, 2]. In addition, Cd levels are higher in biological samples such as blood, hair, and dental calculus in smokers than in nonsmokers [10–12]. In this study, Cd was not detected in the three participants who did not use tobacco products. However, the Cd level in the OKM of nonsmokers was higher than that of smokers, although the difference was not statistically significant. This result primarily suggests that the OKM does not exhibit the characteristic cumulative accumulation of heavy metals and toxic/carcinogenic elements in the hair and calculus.

**Fig. 3:**
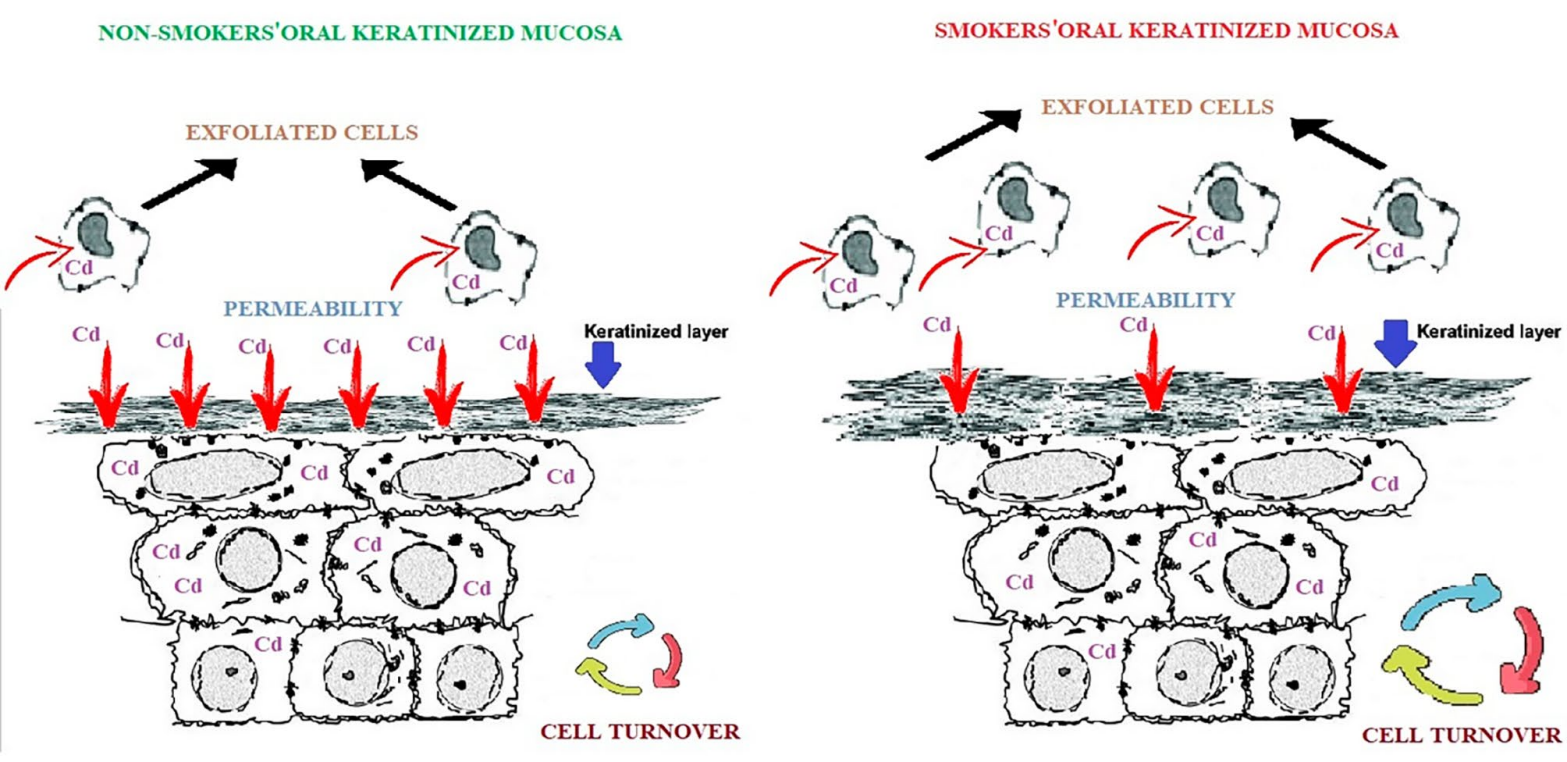
Schematic representation of possible changes affecting cadmium accumulation in the oral keratinized mucosa due to tobacco use

In addition to tobacco products, the consumption of contaminated food/seafood and environmental pollution are important sources of Cd exposure [1–3]. The small amount of Cd detected in the OKM of tobacco users suggests that Cd transmission to the oral mucosa through food may be higher than that through tobacco products. However, when the nontobacco Cd exposure (food consumption, industry/environmental pollution exposure, etc.) of the participants were compared, no significant difference was found between the control and study groups. The lower Cd concentration in tobacco users compared with those in nonsmokers can be explained by the effect of tobacco-induced oral mucosal changes (Fig. 3).

The apoptotic mechanism in the oral epithelium is induced by tobacco use [9, 10]. Aged and/or damaged cells are eliminated by apoptosis [17]. The elimination of apoptotic cells caused by the chemical components in tobacco products or the thermal effect of cigarette smoke in tobacco users may have played a role in the detection of less Cd in smokers.

The oral mucosa sends dead cells to the oral cavity by exfoliation [18]. The finding that the number of micronuclei in the exfoliated buccal mucosa of smokers was higher than that of nonsmokers was presented as evidence of tobacco-induced degeneration on the exfoliated epithelium. Considering that the remaining mucosa is free of toxic effects, the lower Cd levels in the oral mucosa of smokers compared with those in nonsmokers can be explained from this opinion [16]. However, because no finding was related to the exfoliated epithelium in the present study, new studies are needed to support this opinion. In addition, studies on the exfoliated epithelium include cells outside the cell cycle, and overestimated results may be obtained because of cumulative accumulations [19].

The mucosal epithelium is involved in the structure of many organs in the body, and changes in epithelial turnover are followed to explain the pathological mechanisms [20]. Changes in the turnover in the oral mucosal epithelium have also been evaluated [21, 22]. A balance relationship was found between apoptosis and cell turnover, and changes in cell turnover were interpreted by monitoring apoptosis in OSCC, oral submucous fibrosis, and oral leukoplakia [23]. The cell turnover rate varies differently in two different conditions that have similar autoimmune mechanisms, such as lichen planus and psoriasis [24]. Although the OKM has not shown any histological changes such as dysplasia or hyperkeratosis, cell turnover changes may occur primarily via direct contact with tobacco products. It is not possible to comment on the direction of change (decrease/increase) of the cell cycle with the data obtained in this study. However, the finding that Cd was higher in smokers in dental calculus, which includes components that show cumulative accumulation such as dead cell residues and where there is no cell cycle, may support the effect of cell turnover on Cd accumulation [12, 24]. The positive correlation between age and Cd levels can also be explained by the decrease in the oral epithelial turnover rate over time.

The use of tobacco products plays an important role in the formation of mucosal white lesions such as oral leukoplakia, and hyperkeratosis is one of the common morphological changes in oral leukoplakia [13, 14, 25]. In individuals who smoke but do not show epithelial dysplasia or hyperkeratosis and have not been diagnosed with oral leukoplakia, a microscopic increase in the keratin layer may be possible. This situation can be defined as an increase in the protection task developed by the oral mucosal epithelium against toxic and carcinogenic stimuli. Similarly, the finding that the nonkeratinized oral mucosa is more permeable than the keratinized oral mucosa may be a result of this protective function [26]. Despite the lack of macrolevel pathology such as leukoplakia in the OKM in smokers, the change in keratinization and permeability may play a role in the lower Cd level in smokers.

Cd is one of the factors involved in the development of oral cancer [8–10]. The results of this study cannot reveal that Cd plays a dominant role in oral cancer development. A cell line study can be planned to compare the physiological and biological changes of cells exposed to tobacco products (study group) and those exposed to Cd-free tobacco products (control group) to demonstrate the main role of Cd in oral cancer development.

The study participants were asked about the frequency of consumption of foods likely to contain Cd and the possibility of occupational or environmental exposure, except for the habit of using tobacco products. However, the density of elements with which Cd is metabolically competitive, such as Zn and Fe, may also have influenced the amount of Cd detected in the OKM [3]. However, reliable assessment of the levels of elements such as Zn and Fe was not possible in the present study because they can be taken in different concentrations through drugs, nutritional supplements, or various natural foods. In future studies, the levels of these trace elements can be determined with additional samples, such as blood obtained from patients, and their correlation with the Cd level can be examined.

Because a standard punch biopsy apparatus was used to take the samples, OKM weights may be kept equivalent to the mucosal thickness. However, this opinion cannot be supported because no histological analysis was performed on the study samples. The finding that the Cd level in the OKM is higher than that in women and the decrease in the OKM weight in smokers compared with those in women suggests that men are more sensitive to Cd accumulation in the OKM and that their oral mucosa is more prone to erosive changes.

In this study, only Cd concentrations in the OKM were measured. Procedures to reveal cellular mechanisms and abnormal changes, such as histological analysis (changes in mucosal thickness, keratinization differences, etc.) or metabolic marker detection were not performed on the study samples. Tobacco products contain many toxic elements and chemicals, except for Cd, and the complex content of all chemicals in tobacco products is responsible for pathological changes. New studies are required to determine the isolated biological effects of elemental ingredients such as Cd in tobacco. In future studies, both Cd concentration and histological examination can be evaluated together in OKM samples. However, it may not be appropriate to perform both Cd concentration measurements and histological examination with the same sample. Because the sample is exposed to various laboratory chemicals during the first process (concentration determination or histological analysis), the results of the other process may be suspicious. Biopsy samples with suspicion of pathology were not included in this study because the detection of Cd concentration by the OKM processed in histological examination may not be reliable. Prioritizing Cd concentration analysis in samples taken for biopsy or obtaining these samples in accordance with Cd concentration analysis may cause ethical violations in clinical practice. This problem can be overcome by taking more than one sample (if more than one implant is applied) from the participants in the study.

ICP mass spectrometry and atomic absorption spectrometry can also be used to determine the Cd concentration [8]. The results may vary depending on the chosen analysis technique and procedure. In addition, the collection of the study samples from a single dental clinic can be a limitation. Cd concentration studies using different analysis methods and multicenter participation may provide new findings in demonstrating the effect of tobacco on nonkeratinized mucosa.

## Conclusions

The finding that the Cd concentration in the OKM taken from smokers was lower than that in nonsmokers suggests that the OKM does not have a cumulative accumulation characteristic in terms of toxic elements. Changes in the turnover rate, keratinization, and apoptotic mechanisms in the OKM due to the thermal/chemical effect of tobacco may be also responsible for the difference in Cd accumulation. Studies that include histological analyses, evaluate elemental food supplements, and offer wider participation are needed to comprehensively examine Cd-induced OKM changes.

## Data Availability

The datasets generated and/or analyzed during the current study are not publicly available due to their lack of systematic order, but are available from the corresponding author upon reasonable request.
